# Performance Improvement of Steel Fiber Reinforced High-Performance Concrete Cured by Electric-Induced Heating Under Negative Temperature by Mix Proportion Optimization

**DOI:** 10.3390/ma18102231

**Published:** 2025-05-12

**Authors:** Yishu Zhang, Han Wang, Wei Wang

**Affiliations:** 1 School of Civil Engineering, Harbin Institute of Technology, Harbin 150090, China; 2 School of Infrastructure Engineering, Nanchang University, Nanchang 330031, China

**Keywords:** electric-induced heating curing, high-performance concrete, curing temperature, mechanical strength

## Abstract

To address the insufficient early strength development of steel-fiber-reinforced high-performance concrete (SF-HPC) under subzero temperatures, this study proposes an electric-induced heating curing method for SF-HPC fabrication at −20 °C. The effects of mix parameters, including steel fiber content, water-to-binder ratio, silica fume dosage, and fine aggregate gradation, on the curing temperature and mechanical properties of SF-HPC were systematically investigated. The optimal mix proportion was identified through the curing temperature and compressive strength development for the specimens. Results revealed that compressive strength initially increased and then decreased with higher silica fume content and fine aggregate replacement ratios, while increased water-to-binder ratios positively influenced curing efficiency and strength development. The optimal mix comprised 2.0 vol% steel fibers, a water-to-binder ratio of 0.22, 20% silica fume, and 60% fine aggregate replacement. Further, comparative analyses of electric-induced heating curing, room-temperature curing, and high-temperature steam curing revealed that electric-induced heating curing can promote the strength formation of SF-HPC in a negative-temperature environment. Microstructural characterization via BET analysis demonstrated that electric-induced heating curing refined the pore structure of SF-HPC. These findings highlight the benefits of electric-induced heating as an efficient strategy for fabricating SF-HPC in cold climates, providing theoretical and practical insights for winter construction.

## 1. Introduction

The construction industry in cold regions faces challenges due to the harsh environmental conditions that impede conventional concrete curing processes [1,2]. Subzero temperatures retard hydration reactions, leading to delayed strength development, prolonged construction cycles, and compromised durability of concrete structures [3,4]. Traditional curing methods, such as external heating or insulation, often prove inefficient in extreme cold climates, as they struggle to maintain uniform thermal conditions and consume substantial energy resources. Even if they can be used in negative-temperature environments, the environmental temperature is restricted to not be lower than −10 °C to ensure a promising curing quality [5,6,7]. Moreover, the formation of ice within the concrete matrix during early-age freezing can induce internal stresses, resulting in microcracking and reduced long-term performance [8,9]. Under this circumstance, it is of importance to find a novel curing regime for concrete construction under severely low temperatures.

The utilization of fiber-reinforced concrete (FRC) in cold regions has gained significant attention due to its enhanced durability under freeze–thaw cycling and deicing salt exposure. Incorporating discrete fibers (steel, polypropylene, or basalt) at a 0.5–2.0% volume fraction effectively mitigated thermal-stress-induced cracking through crack-bridging mechanisms. To promise a higher performance to resist the harsh environment in cold regions, high-performance concrete (HPC), characterized by its ultra-dense microstructure, low permeability, and exceptional mechanical resilience, presents a compelling solution for infrastructure projects in harsh environments [10,11,12]. Through the incorporation of supplementary cementitious materials (SCMs) like silica fume, fly ash, and slag, along with optimized water-to-binder ratios and superplasticizers [13,14,15], HPC achieves superior compressive strength (>100 MPa) and resistance to chemical aggression [16,17,18]. However, the very attributes that render HPC advantageous under standard conditions become liabilities in cold climates. The reduced water content and highly packed particle matrix limit the availability of free water for hydration, while low ambient temperatures further suppress reaction rates, resulting in prolonged setting times and insufficient early-age strength [19,20,21]. Compounding these issues, the introduction of steel fibers—a common strategy to enhance fracture toughness and ductility—introduces new complexities. Although steel fibers effectively bridge microcracks and improve post-cracking behavior, their uneven dispersion within the cementitious matrix can create localized weak zones, undermining both mechanical performance and functional characteristics such as electrical conductivity [22,23]. This paradox underscores a critical challenge: while HPC is theoretically ideal for demanding cold-region applications, conventional curing approaches fail to reconcile its material potential with the thermodynamic constraints imposed by subfreezing environments.

Electrically assisted curing methods, particularly electric-induced heating techniques, have garnered increasing attention as a paradigm-shifting approach to address these limitations [24,25]. By embedding conductive elements such as steel fibers or carbon-based materials within the concrete matrix, researchers can harness electrical resistance heating to generate internal thermal energy, creating a self-curing system that actively counteracts ambient cold [26,27]. This method offers distinct advantages over external heating approaches, including energy efficiency, spatial uniformity of temperature distribution, and compatibility with automated control systems [28,29]. Crucially, electric-induced heating curing synergizes with the intrinsic properties of steel-fiber-reinforced HPC—the fibers not only serve as reinforcement but also form percolating conductive networks that enable efficient electrical–thermal conversion. Recent studies have demonstrated that elevated curing temperatures (≥40 °C) achieved through electric-induced heating can accelerate pozzolanic reactions, enhance early-age strength development, and refine pore structures even in low-temperature environments [24,30]. Nevertheless, the practical implementation of this technology remains constrained by insufficient understanding of how material composition and processing parameters govern the behavior of the composite system. Key knowledge gaps persist regarding the optimization of conductive filler content, the interplay between binder chemistry and electrical conductivity, and the effects of mixture proportioning on both curing efficiency and final material properties.

In this work, electric-induced heating curing was used to prepare steel-fiber-reinforced high-performance concrete (SF-HPC) at −20 °C. The specific effect of the mix proportion on the performance of electric-induced heating cured SF-HPC was clarified including the steel fiber content, water-to-binder ratio, silica fume content, and fine aggregate gradation. Once the optimal SF-HPC mix proportion was determined, electric heating curing was conducted, and the performance of SF-HPC was compared with that of the specimens cured by room-temperature curing and high-temperature steam curing. The hydration degree and pore structure of the specimens were further investigated to disclose the benefits of electric-induced heating curing as a negative-temperature concrete curing method.

## 2. Materials and Methods

### 2.1. Raw Materials

P.O. 42.5 cement produced by Yatai Ltd., Harbin China (Tian et al., 2024) [27] and silica fume were used as the main cementitious materials for the preparation of SF-HPC. The main chemical composition and physical properties of the cement are listed in Table 1 and Table 2. Silica sand with a diameter of 0.84–2 mm was used as the fine aggregate. The diameter and length of steel fiber were 0.22 mm and 13 mm, respectively. There is no bar reinforcement in the specimen.

### 2.2. Test Methods

#### 2.2.1. Electrical Resistance Measurement

The two-electrode testing method was used in this work to measure the electric resistance of the SF-HPC specimens, in which two electrodes were inserted at both ends of the specimen. This method was simple to operate, and the electrode placement method facilitates subsequent mechanical property testing. A Tonghui TH2810D high-frequency digital LCR meter (Tonghui Ltd., Changzhou, China) was used as the test equipment (Ouyang et al., 2022) [21], and the test frequency was set to 10 kHz, which can effectively eliminate the interface resistance between the electrodes and the specimen. Under this circumstance, the resistance measured by the LCR meter can be regarded as the actual resistance of the specimen. Further, the electric resistivity of the specimen was then calculated using Equation (1) based on the measured resistance values (Tian et al., 2024) [28].

(1) $$\rho =\frac{RS}{d}$$

Here, *ρ* is the electric resistivity of the specimen, *S* is the contact area between the electrode and the specimen, *d* is the distance between the electrodes, and *R* is the measured electric resistance of the specimen.

#### 2.2.2. Temperature Measurement

For SF-HPC cured by electric-induced heating in a negative-temperature environment, the curing temperature determines the performance of the specimens. Therefore, real-time monitoring of the specimen’s curing temperature is required during experiments. Specifically, a multi-channel temperature data logger was used to measure the temperature of SF-HPC specimens during the curing process, and the thermocouple was placed at the center of the specimen. To prevent the electric current flow through the thermocouple during the curing process from interfering with temperature recording, the thermocouple was insulated by adhesive tape. During the experimental process, the temperature data logger was configured to record the central temperature of the specimen every 30 s.

#### 2.2.3. Mechanical Strength Test

SF-HPC specimens with a size of 40 mm × 40 mm × 40 mm were used for the compressive strength test, and the test procedure was referenced from ASTM C109/C109M-11. The loading rate was 2.4 ± 0.2 kN/s during the test process, and a total of six specimens were tested for each batch for reliable results.

### 2.3. Curing Regimes

An electric heating curing regime was used to ensure the strength formation of SF-HPC under severely low temperature. Room-temperature curing at 20 ± 2 °C and humidity of >95% was conducted for comparison, as well as high-temperature steam curing at 60 °C and humidity of >95%. To be more specific, a 12 h electric heating curing regime was conducted to determine the effect of various factors on the performance of the samples, and a 2-day electric heating curing regime was employed to prepare the sample with optimized mix proportions.

### 2.4. SF Content Determination

Among the multiple factors, the determination of SF contents is of significance, because it is the foundation for the continuous electric-induced heating curing process. Excessive steel fiber content adversely affects the fluidity of the mixture. Therefore, steel fiber content in concrete structures should not be overly high. However, when steel fiber content is too low, the internal steel fibers in high-performance concrete become insufficient to form fully interconnected conductive pathways, leading to excessively high initial resistivity that hinders electrical energy supply. Consequently, it is essential to determine an optimal steel fiber dosage to ensure a successful electric-induced heating curing process and facilitate the effective preparation of SF-HPC in low-temperature environments. In this work, the optimal SF contents were determined based on the experimentally measured percolation threshold, taking the initial resistivity as the judgement standard.

### 2.5. Microstructural Characterization

The pore structure development of SF-HPC specimens cured by different methods was disclosed by Brunner–Emmet–Teller (BET) measurement. The specimens were broken into small pieces and immersed in ethanol to stop the hydration reaction. Then, a 48 h drying operation was conducted, and the specimens were degassed using a vacuum at 80 °C for 6 h, and nitrogen (N_2_) was the adsorbate.

## 3. Results and Discussion

The optimal SF content was initially determined to provide the foundation for the implementation of electric-induced heating curing. Subsequently, the specific effects of each component on the performance of electric-induced heating cured specimens were clarified based on the heating and mechanical test results.

### 3.1. Determination of SF Contents

The mix proportions to determine the SF contents are listed in Table 3. As this experiment focused on determining steel fiber content as a foundational study, factors such as silica fume and quartz sand gradation were not considered in the mix design. Specimens were prepared using conventional concrete mixing procedures: cement and quartz sand were first poured into a mixing pot and rapidly stirred for 2 min, water and superplasticizer were then added and rapidly mixed for 3 min, then steel fibers were incorporated and rapidly stirred for 5 min.

**Figure 1:**
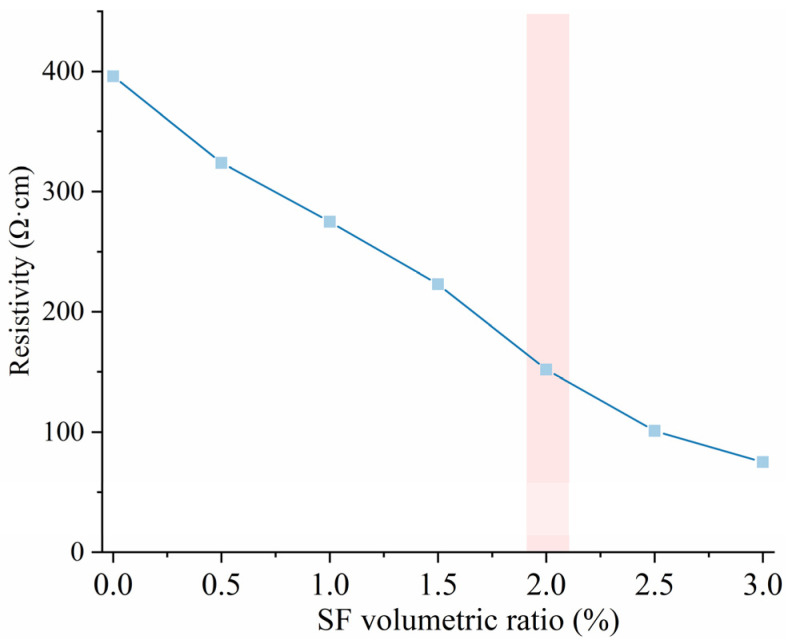
Initial resistivity change with different SF contents.

Figure 1 depicts the initial resistivity change of SF-HPC with various SF contents. It can be found that the specimens without steel fibers exhibited high initial resistivity (around 400 Ω·cm), indicating poor electrical conductivity of the specimen that was not suitable for electric-induced heating curing, especially for the experiments conducted under subzero temperature conditions. However, as steel fiber content gradually increased, the initial resistivity of the fresh mixture progressively decreased. When steel fiber content reached 2.0 vol%, the initial resistivity dropped to 152 Ω·cm, representing a 61.6% reduction compared to the specimen with no SFs. A further increase in SF content can induce the reduction of resistivity to a certain degree, while the resistivity for the samples with 2.5 vol% SF contents was still above 100 Ω·cm. Considering the resistivity of around 150 Ω·cm, the experimentally determined percolation threshold for SFs forming a fully connected conductive pathway was confirmed as 2 vol% after taking the economic cost into consideration.

### 3.2. Effect of Silica Fume Content on Curing Temperature and Compressive Strength

This section mainly studies the influence of different silica fume contents on the curing temperature and mechanical properties of SF-HPC cured by electric-induced heating in a −20 °C environment. The experiment was divided into four groups. The addition method of silica fume was the external addition method, and the external content accounted for 10 wt%, 15 wt%, 20 wt%, and 25 wt% of the cement mass, respectively. The water–binder ratio of the specimen was fixed at 0.25, and the cement–sand ratio was 1:1. The specific experimental mix design is shown in Table 4. During the curing process, the electric power of electric-induced heating curing was maintained at a constant 15 W, and the curing period of this experiment was 12 h. After reaching the set curing age, the specimens were taken out and placed in a standard curing environment for 36 h. Then the compressive strengths SF-HPC specimens were tested to determine the optimal addition amount of silica fume.

**Figure 2:**
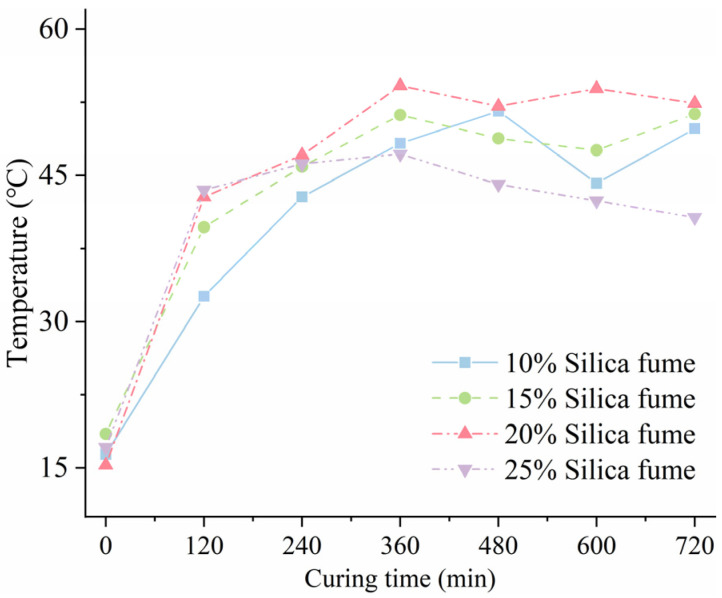
Curing temperature of SF-HPC with various silica fume contents.

Figure 2 depicts the temperature development of SF-HPC specimens with different silica fume contents during the electric-induced heating curing process at −20 °C. It can be observed from Figure 2 that, in the heating stage, with the increase in silica fume contents, the slope of the curing temperature growth of the specimen increased first then decreased. When the silica fume content was 20 wt%, the temperature of the electric-induced heating cured specimens increased the fastest, and the temperature rise slope was the largest. This may be because an excessive amount of silica fume may have an impact on the fluidity of the matrix at subzero temperatures. In the constant-temperature stage, the temperature of the specimens with 15 wt % and 20 wt % silica fume can be stabilized above 50 °C, and the specimens with 20 wt % silica fume have better temperature stability. However, with the increase in silica fume content, the specimens with 25 wt % silica fume showed very poor temperature stability. It can even be said that, under such conditions of silica fume content, the specimens do not have stable heating ability, which seriously affects the efficiency of OH curing of specimens in subzero-temperature environments. This was also considered to be the effect of excessive silica fume content on the fluidity of the specimens.

**Figure 3:**
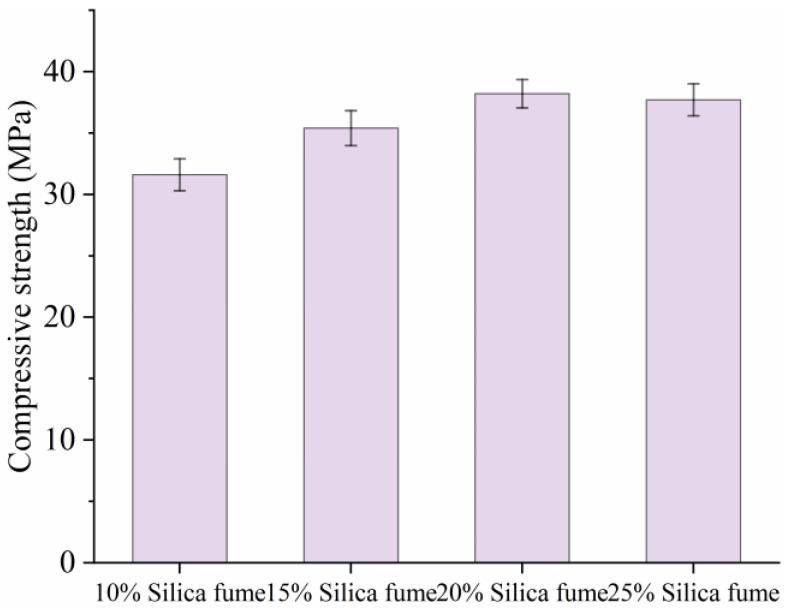
Compressive strengths of SF-HPC with various silica fume contents.

Figure 3 depicts the compressive strengths of SF-HPC with various silica fume contents cured by 12 h electric-induced heating curing at −20 °C. It can be seen from Figure 3 that, similar to the law of temperature development, with the increase in silica fume content, the mechanical properties of the specimens first showed an increasing trend and then a decreasing trend. The specimens with 10~25 wt% silica fume contents had values of 31.6 MPa, 35.4 MPa, 38.2 MPa, and 37.7 MPa, respectively. The highest compressive strength can be found in the specimen incorporating 20 wt% silica fume. With an increasing silica fume content, the decrease in strength of OH curing specimens may be attributed to two reasons. On the one hand, as shown in Figure 2, the curing temperature of specimens with 25 wt% silica fume during electric-induced heating curing in a subzero-temperature environment was lower than that of specimens with 20 wt% silica fume, which had an adverse effect on the development of the compressive strength of specimens. On the other hand, when the content of silica fume was too high, it will compete with the cement inside the specimen, resulting in a lower amount of hydrated cement. Based on the experimental results, the optimal silica fume content was determined to be 20 wt%.

### 3.3. Effect of Sand Gradation Design on Curing Temperature and Compressive Strength

This section mainly studies the influence of the ratio of coarse sand and fine sand on the curing temperature and compressive strength of SF-HPC at −20 °C. The experiment was divided into five groups, and the proportions of fine sand replacing coarse sand were 20%, 40%, 60%, 80%, and 100%, respectively. To be more specific, the particle size range of coarse sand was 1.5~2 mm, and the particle size range of fine sand was 0.84~1.5 mm. Based on the above two groups, the volume content of steel fiber was 2 vol%, and the silica fume content was 20 wt%. In this section, the water–binder ratio was fixed at 0.25. The specific mix ratio is shown in Table 5 below.

**Figure 4:**
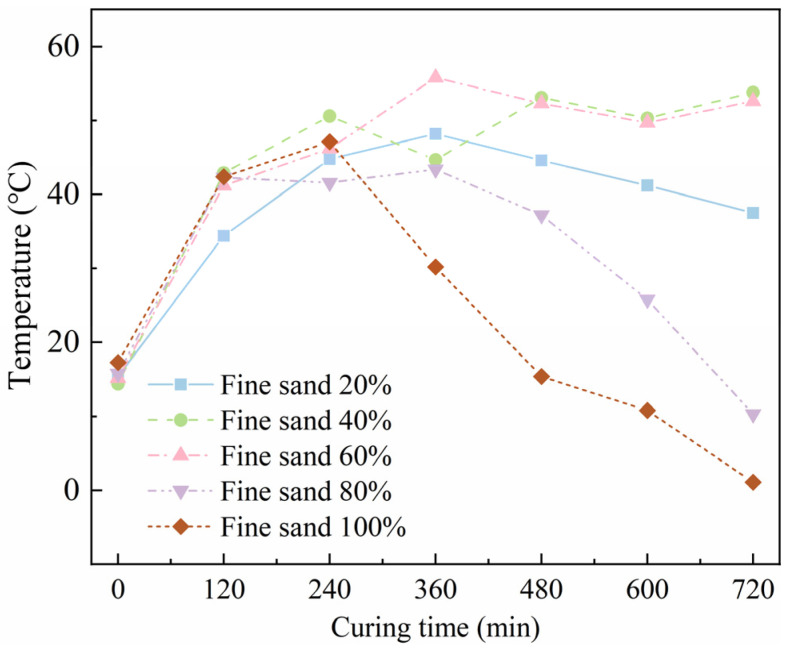
Temperature development of SF-HPC with various fine sand ratios.

Figure 4 shows the temperature development curve of SF-HPC with different fine sand ratios during the electric-induced heating curing process at −20 °C. It can be seen from Figure 4 that, during the electric-induced heating curing process, the sand gradation affected the curing temperature of the specimens. When the gradation was poor, such as when the proportion of fine sand replacing coarse sand was too low or too high, the maximum temperature was low, especially when the replacement amount of fine sand was 100%. The temperature development of the specimen was negatively affected, and the temperature was near zero in the final stage of electric-induced heating curing, as the electric resistance was too high, and constant electric power was hard to achieve. Moreover, when the replacement rate of fine sand reached 40% or 60%, the electric-induced heating curing specimen exhibited the highest curing temperature and, when the fine sand ratio was higher than 60%, the curing process was obviously influenced with poor curing efficiency.

**Figure 5:**
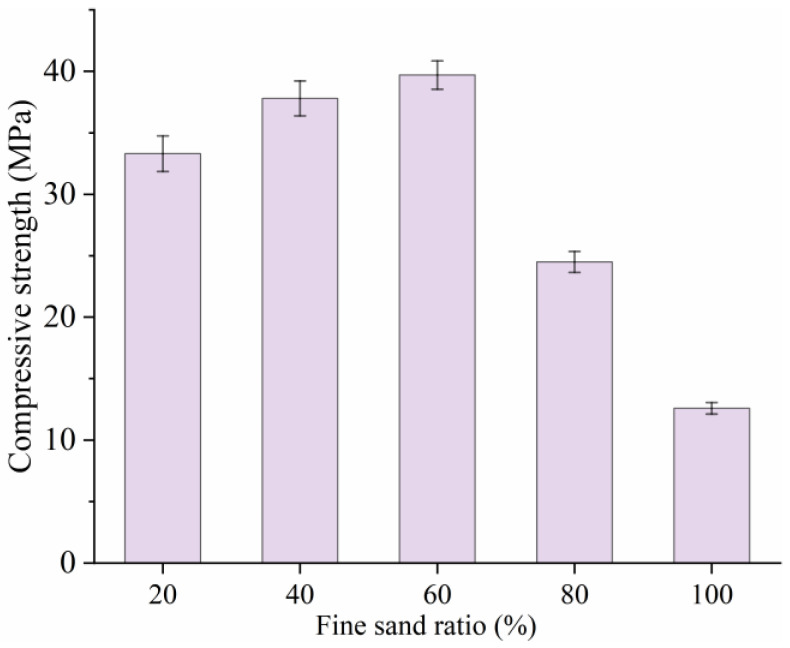
Compressive strength of SF-HPC with different fine sand replacement ratios.

For the electric-induced heating cured SF-HPC, a higher fine sand ratio was beneficial for the strength improvement of the specimen, as depicted in Figure 5. To be more precise, the 12 h compressive strengths of the specimens with different fine sand replacement rates were 33.3 MPa, 37.8 MPa, 39.7 MPa, 24.5 MPa, and 12.6 MPa, respectively. It can be seen from the results that, when the replacement rate of fine sand was 40% and 60%, the compressive strengths of electric-induced heating cured SF-HPC were above 35 MPa, which was improved compared with the specimens with replacement rates of fine sand of 20%, 60%, and 100%. This shows that a proper increase in the replacement rate of fine sand was beneficial to its mechanical properties and a high replacement rate of fine sand can play a role in filling pores for high-performance concrete and thus promote the improvement of mechanical properties. The reduced compressive strength was consistent with the temperature results, that, under the condition of high fine sand replacement rate, the electric-induced heating cured specimen cannot undergo smooth curing under subzero-temperature conditions, which affects the formation of structural strength. The reason for this phenomenon may be that, when the replacement rate of fine sand was 100%, the size of fine aggregate was too small, and the water absorption effect was too obvious, which seriously affected the fluidity of the matrix and hindered the uniform dispersion of steel fiber in the matrix. Moreover, too much water absorption will also lead to the reduction of water for hydration. Above all, the optimal fine sand replacement ratio was determined to be 60%.

### 3.4. Effect of Water-to-Binder Ratio on Curing Temperature and Compressive Strength

For HPC composite, the water-to-binder ratio is a key factor affecting the performance of the specimen, as it influences the hydration reaction and material density. This section mainly studies the change in temperature and compressive strength of electric-induced heating cured SF-HPC in the early curing process at −20 °C under different water–binder ratios. The experiment was divided into five groups, and the water–binder ratio was set to 0.16, 0.18, 0.2, 0.22, and 0.24, respectively. The specific mix proportion is shown in Table 6 below.

**Figure 6:**
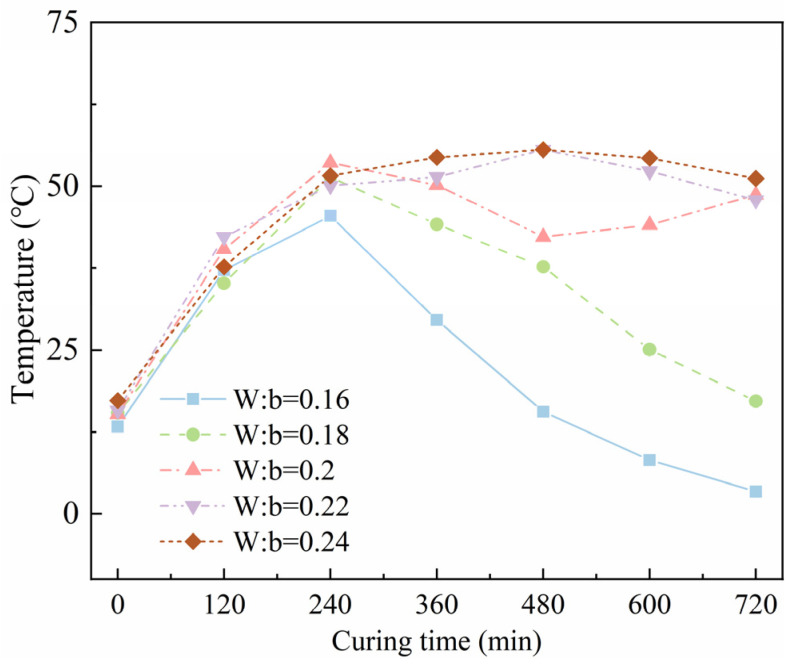
Temperature development of SF-HPC with various water-to-binder ratios.

Figure 6 illustrates the temperature development of SF-HPC with different water-to-binder ratios during the electric-induced heating curing process at −20 °C. It can be seen that the temperature–time curves of SF-HPC specimens with different water-to-binder ratios can be divided into three stages: heating stage, constant-temperature stage, and cooling stage. In the heating stage, the slope of the temperature curve was higher as the water-to-binder ratio increased, which may be because a lower water-to-binder ratio may not be suitable for a continuous electric-induced heating process, thus hindering the curing temperature of the specimens. This reflected that the water-to-binder ratio showed a certain influence on the curing quality of the specimen. To be more specific, the conductive phases inside SF-HPC specimens included conductive ions inside the pore solution and steel fiber, and the curing temperature of SF-HPC under electric-induced heating curing was closely related to the electric conductivity of the specimen. When the water-to-binder ratio was too low, the effective conductivity paths in the specimen were much reduced, resulting in the low electric conductivity. As the water-to-binder ratio increased, the curing temperature of electric heating cured SF-HPC was much improved to a high level above 50 °C, which was suitable for the strength formation of SF-HPC cured under subzero temperatures.

**Figure 7:**
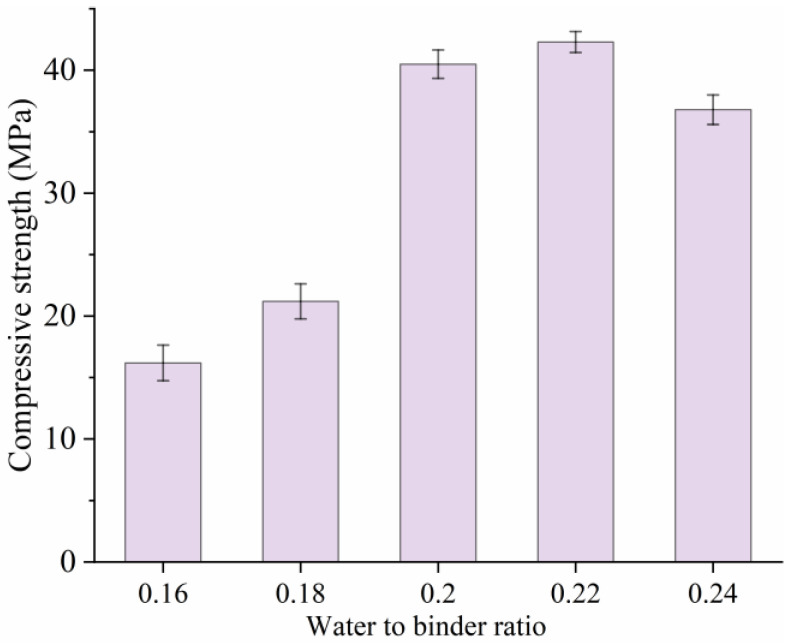
Compressive strengths of SF-HPC with various water-to-binder ratios.

Figure 7 depicts the compressive strength development of electric-induced heating cured SF-HPC with various water-to-binder ratios. It can be seen that with the increase in water-to-binder ratio, the compressive strengths of the samples were much improved, and this was because the low curing temperature for the specimens with the water-to-binder ratios of 0.16 and 0.18 cannot perform the rapid strength formation of SF-HPC, and the higher curing temperature in the specimens with higher water-to-binder ratios contributed to the strength improvement. The compressive strength results seemed different from the literature showing that a lower water-to-binder ratio negatively influenced the compressive strength for the specimens but, considering the novel curing system for the material, it is understandable. And the optimal water-to-binder ratio was determined to be 0.22 in this work.

To sum up, according to the curing temperature and compressive strength test results, the determined mix proportion for SF-HPC was determined as listed in Table 7.

### 3.5. Comparison Between Different Mix Proportions

**Figure 8:**
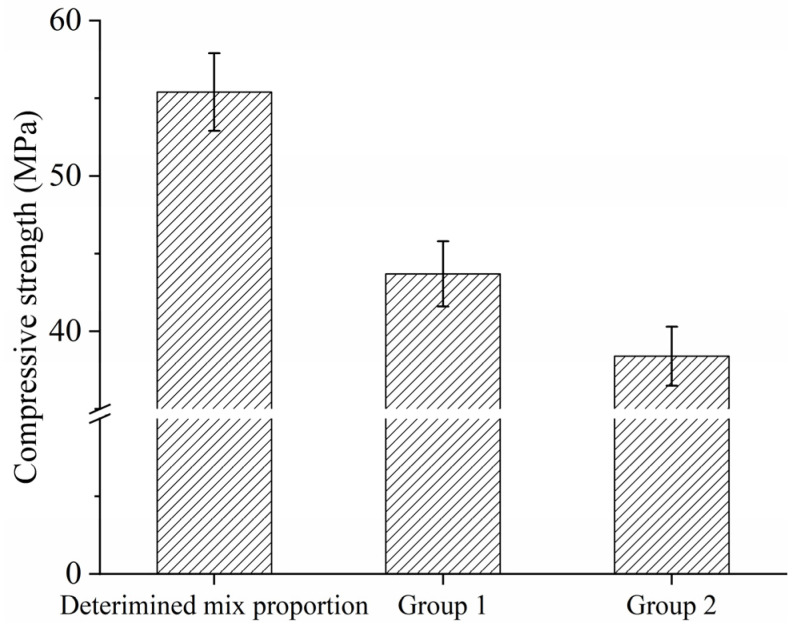
Compressive strengths of the specimens with different mix proportions.

To prove the effectiveness of the determined mix proportion for electric-induced heating cured SF-HPC, two other mix proportions were taken as examples for comparison. In Group 1, the silica fume content, fine sand replacement ratio, and water-to-binder ratio were 15 wt%, 40%, and 0.22, respectively; in Group 2, the silica fume content, fine sand replacement ratio, and water-to-binder ratio were 10 wt%, 40%, and 0.18, respectively. Two-hour electric-induced heating curing was conducted to prepare the specimens with an environmental temperature of −20 °C, and the compressive strength results are exhibited in Figure 8. It can be found from Figure 8 that the compressive strengths of Group 1 and Group 2 were much lower than that of the determined mix proportion, indicating the above experiments were helpful to pre-determine the optimal silica fume, water-to-binder ratio, and fine aggregate replacement ratio. Under this circumstance, the determined mix proportion was further used to evaluate the effectiveness of the curing regime.

### 3.6. Implementation of Electric-Induced Heating Curing on SF-HPC with Optimal Mix Proportion

To evaluate the effectiveness of electric-induced heating curing in the preparation of SF-HPC in a severely cold environment, RT curing and HTS curing were conducted for the comparison. The curing duration of electric-induced heating was set to 2 days with the curing temperature concentrated in the range of 57–62 °C, and the curing duration for RT curing was 3 days, which became 2 days for HTS curing.

#### 3.6.1. Compressive Strength

**Figure 9:**
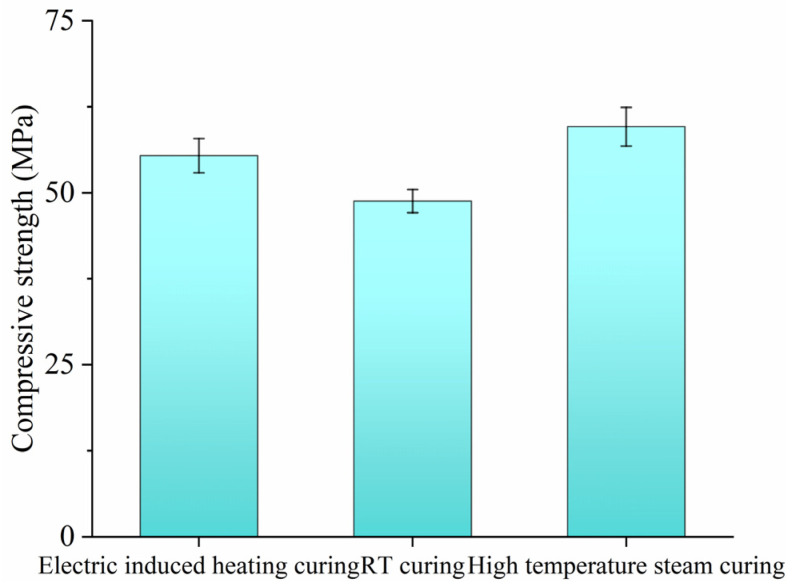
Compressive strengths of SF-HPC cured by various methods.

Figure 9 depicts the compressive strength of SF-HPC cured by different methods. It can be found that electric-induced heating curing improved the compressive strength of SF-HPC under −20 °C of 55.4 MPa with the curing age of 2 days, which was much higher than that of the specimen cured by 3 days’ RT curing (48.8 MPa), showing an increase of 13.5%, and this was also consistent with the strength of the specimen cured by 2 days’ high-temperature steam curing. The compressive strength highlighted the advantage of electric-induced heating for fabricating high-performance concrete in a severely cold environment.

#### 3.6.2. Pore Structure Analysis

**Figure 10:**
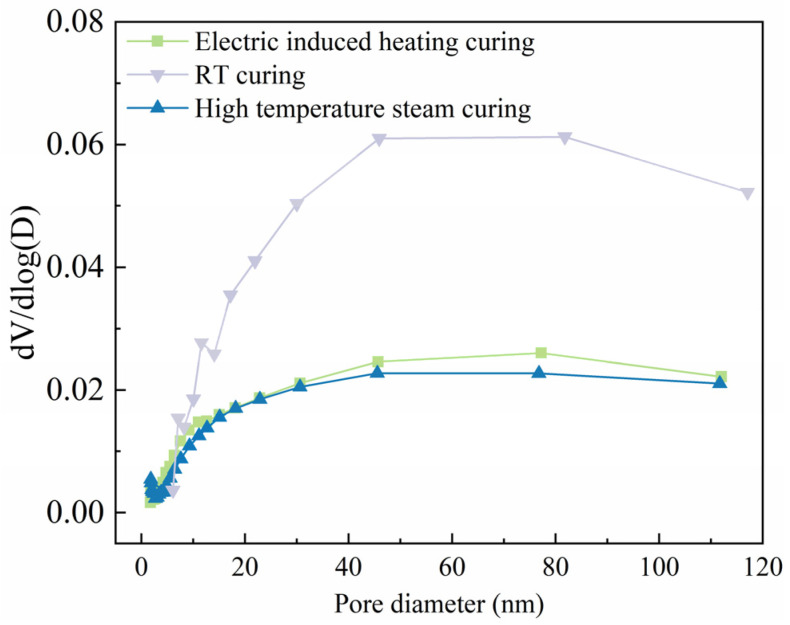
BET pore structure analysis of SF-HPC specimens cured by different methods.

BET analysis was conducted to investigate the pore structure of SF-HPC specimens cured by different methods as exhibited in Figure 10. RT cured specimens depicted the largest pore distribution situation, while the specimens cured by electric-induced heating curing and high-temperature steam curing depicted comparable pore size distributions, indicating the similar pore structure inside the specimens. The BET analysis results reflected the advantage of electric-induced heating curing for refining the pore structure of SF-HPC even in a severely cold environment.

## 4. Conclusions

This study demonstrated the efficiency of electric-induced heating curing as a novel method to overcome the challenge of insufficient early strength development in steel-fiber-reinforced high-performance concrete (SF-HPC) fabricated at subzero temperatures (−20 °C). Through systematic investigation of mix parameters, including steel fiber content (0–3.0 vol%), water-to-binder ratio (0.16–0.24), silica fume dosage (10–25%), and fine aggregate replacement ratio (0–100%), the optimal SF-HPC mix proportion was determined by evaluating curing temperature and compressive strength. Experimental results delineated that the compressive strength of electric-induced heating cured specimens showed an increasing trend and then a decreasing trend as the silica fume content and fine aggregate replacement ratio increased. Moreover, the increased water-to-binder ratio illustrated a positive effect on the curing temperature and strength development for the specimens. The optimal steel fiber content was 2.0 vol%, the water-to-binder ratio was 0.22, and the silica fume content was 20% with a fine aggregate replacement ratio of 60%. Comparative analysis with conventional room-temperature curing and high-temperature steam curing highlighted the superiority of electric-induced heating curing in promoting early-age mechanical performance, attributed to its uniform heating effect that mitigated low-temperature inhibition on cement hydration. BET analysis further proved that electric-induced heating curing refined the pore structure of SF-HPC. This work provides new insights into high-quality concrete construction in cold environments.

## Figures and Tables

**Table 1:** Chemical composition of cement (wt%).

Oxide	SiO_2_	Al_2_O_3_	Fe_2_O_3_	MgO	CaO	SO_3_
Content	22.34	4.23	3.05	1.45	63.18	2.22

**Table 2:** Physical properties of cement.

Setting Time (min)		Compressive Strength (MPa)		Flexural Strength (MPa)	
Initial setting	Final setting	3 d	28 d	3 d	28 d
185	239	16.9	46.6	4.8	7.5

**Table 3:** Mix proportion design for SF content determination.

Water-to-Binder Ratio	Binder-to-Sand Ratio	SF Contents (vol%)	PS (wt%)
0.25	1:1	0	0.5
0.25	1:1	0.5	0.5
0.25	1:1	1	1
0.25	1:1	1.5	1
0.25	1:1	2	1
0.25	1:1	2.5	1.5
0.25	1:1	3	2

**Table 4:** Mix proportion design.

Binder:Sand	Water:Binder	SFs (vol%)	PS (wt%)	Silica Fume Content (wt%)
1:1	0.25	2	2	10
1:1	0.25	2	2	15
1:1	0.25	2	2	20
1:1	0.25	2	2	25

**Table 5:** Mix proportion design.

Silica Fume Content (wt%)	Binder:Sand	Water:Binder	SFs (vol%)	Fine Sand Ratio	PS (wt%)
20	1:1	0.25	2	20%	2
20	1:1	0.25	2	40%	2
20	1:1	0.25	2	60%	2.5
20	1:1	0.25	2	80%	2.5
20	1:1	0.25	2	100%	3

**Table 6:** Mix proportion design.

Silica Fume (wt%)	Fine Sand Ratio	Water:Binder	SFs (vol%)	PS (wt%)
20	60%	0.16	2.5	3.5
20	60%	0.18	2.5	3
20	60%	0.2	2.5	2.5
20	60%	0.22	2.5	2.5
20	60%	0.24	2.5	2

**Table 7:** Determined mix proportion for SF-HPC.

Silica Fume (wt%)	Fine Sand Replacement Ratio (%)	Water:Binder	SFs (vol%)
20	60	0.22	2.0

## Data Availability

The data presented in this study are available upon request from the corresponding author.
